# High-Accuracy Deep Learning-Based Detection and Classification Model in Color-Shift Keying Optical Camera Communication Systems

**DOI:** 10.3390/s25175435

**Published:** 2025-09-02

**Authors:** Francisca V. Vera Vera, Leonardo Muñoz, Francisco Pérez, Lisandra Bravo Alvarez, Samuel Montejo-Sánchez, Vicente Matus Icaza, Lien Rodríguez-López, Gabriel Saavedra

**Affiliations:** 1 Department of Electrical Engineering, Universidad de Concepción, Edmundo Larenas 219, Concepción 4030000, Chile; franciscavera92@gmail.com (F.V.V.V.); lemunoz2017@udec.cl (L.M.); francisperez@udec.cl (F.P.); gasaavedra@udec.cl (G.S.); 2 Instituto Universitario de Investigación y Desarrollo Tecnológico, Universidad Tecnológica Metropolitana, Ignacio Valdivieso 2409, Santiago 8940000, Chile; 3 Institute for Technological Development and Innovation in Communications, Universidad de Las Palmas de Gran Canaria, 35001 Las Palmas de Gran Canaria, Spain; vicente.matus@ulpgc.es; 4 Facultad de Ingeniería, Universidad San Sebastián, Lientur 1457, Concepción 4030000, Chile; lien.rodriguez@uss.cl

**Keywords:** convolutional neural network (CNN), deep learning, optical camara communication (OCC)

## Abstract

The growing number of connected devices has strained traditional radio frequency wireless networks, driving interest in alternative technologies such as optical wireless communications (OWC). Among OWC solutions, optical camera communication (OCC) stands out as a cost-effective option because it leverages existing devices equipped with cameras, such as smartphones and security systems, without requiring specialized hardware. This paper proposes a novel deep learning-based detection and classification model designed to optimize the receiver’s performance in an OCC system utilizing color-shift keying (CSK) modulation. The receiver was experimentally validated using an 8×8 LED matrix transmitter and a CMOS camera receiver, achieving reliable communication over distances ranging from 30 cm to 3 m under varying ambient conditions. The system employed CSK modulation to encode data into eight distinct color-based symbols transmitted at fixed frequencies. Captured image sequences of these transmissions were processed through a YOLOv8-based detection and classification framework, which achieved 98.4% accuracy in symbol recognition. This high precision minimizes transmission errors, validating the robustness of the approach in real-world environments. The results highlight OCC’s potential for low-cost applications, where high-speed data transfer and long-range are unnecessary, such as Internet of Things connectivity and vehicle-to-vehicle communication. Future work will explore adaptive modulation and coding schemes as well as the integration of more advanced deep learning architectures to improve data rates and system scalability.

## 1. Introduction

Every year, the technology and communications landscape undergoes exponential growth, driven by the increasing demand for high-speed Internet services. However, while this surge in demand emphasizes the need to improve network capacity [1], saturation of the radio frequency (RF) spectrum presents significant challenges [2]. These factors underscore the imperative need to explore new technologies [3] that, although not directly competing with RF in terms of speed, can effectively complement its use in various scenarios [4].

In this context, Optical Wireless Communication (OWC) has emerged as an innovative and viable alternative to the saturated RF spectrum, distinguished by its operation within an unlicensed spectrum and its potential to deliver significantly greater bandwidth than traditional RF systems [5]. Within the realm of OWC, Optical Camera Communication (OCC) technology has experienced substantial technological advancements and renewed research interest. OCC is incorporated in the IEEE 802.15.7 standard [6], which reinforces its feasibility for practical applications and its potential for large-scale adoption in specific environments. This standard introduces Color-Shift Keying (CSK) modulation, employed in this study for its capability to transmit bit streams by varying the colors of a light source.

OCC utilizes hardware from mobile devices to capture video, which acts as a receiver and provides connectivity [7]. Employing light-emitting diodes for data transmission capitalizes on pre-existing infrastructure, thereby significantly reducing deployment costs [8]. Furthermore, OCC derives considerable advantage from continuous advancements in image processing and Deep Learning (DL) technologies to enhance the accuracy and reliability of the receiver [9]. The application of Deep Learning techniques in OCC has been demonstrated to markedly reduce errors in the identification and tracking in receiver devices, even under challenging conditions such as abrupt lighting changes or the presence of obstacles [10].

Several papers in the scientific literature have proposed deep learning-based algorithms to enhance OCC [11,12,13,14]. In [11], an OCC technique for smart factory systems is introduced, which employs an LED array as the transmitter and utilizes On-Off keying modulation. Artificial intelligence is incorporated for LED detection, resulting in a significant improvement in performance compared to traditional methods. By optimizing parameters such as shutter speed, camera focal length, and appropriate channel coding, the system enables stable communication links over distances of up to 7 m. Conversely, ref. [12] proposes the design and implementation of a real-time OCC system capable of operating efficiently under high mobility conditions. For this purpose, the YOLOv8 object detection algorithm is employed, which allows for accurate identification of an LED array used as the emission source. The authors of [13] proposed a display-to-camera optical communication system that uses complementary color barcodes in conjunction with deep neural networks to achieve seamless transmission and reliable communication during normal video playback. This system employs the YOLO model to continuously detect the barcode region on electronic displays and utilizes convolutional neural networks to accurately identify pilot symbols and data embedded in the received images. Furthermore, ref. [14] reports a study on an OCC-based vehicle-to-vehicle communication system using LED arrays as transmitters and cameras as receivers. In addition, other works have addressed the design of OCC systems capable of maintaining reliable communication in dynamic conditions, such as vehicular and underwater environments. For instance, ref. [15] applied deep reinforcement learning to achieve ultra-reliable, low-latency vehicular links, ref. [16] developed a channel-adaptive decoding method for underwater OCC, and [17] employed machine learning to meet uRLLC requirements in vehicular networks.

Faced with the unresolved challenge of ensuring stability and accuracy at practical distances and dynamic conditions in OCC systems, this work proposes an approach based on the efficient use of deep learning that goes beyond detecting signal changes or black-and-white symbols by innovatively leveraging the information contained in color. The main contributions of this paper are as follows:

- The implementation and validation of a novel, high-accuracy deep learning-based receiver architecture for an OCC system. Unlike previous approaches that focus on detecting symbol transitions [18], our proposal uses color as the primary information carrier, focusing on classification of color symbols through the YOLOv8 model, applied for the first time in this context.
- The experimental evaluation over communication distances ranging from 30 cm to 3 m, ensuring the results’ applicability to real-world scenarios and confirming system robustness.
- The integration of advanced data augmentation techniques, including noise addition and overlaying real-world environmental images, to improve robustness and generalization.
- A comprehensive hyperparameter study assessing whether YOLOv8’s default settings are optimal, further verifying the model’s suitability for this specific application.

The remainder of this paper is structured as follows: Section 2 describes the methodology, presenting the proposed Deep Learning-based classifier, detailing the phases of data collection, data preprocessing, model selection and training, as well as validation and adjustment of hyperparameters. Section 3 presents the experiments and discusses the results obtained in the validation of the proposal. Finally, Section 4 summarizes the main conclusions and outlines potential future research directions for further extension.

## 2. Deep Learning-Based Classifier

In this section, the design of the system architecture is presented. The challenges inherent to symbol classification in this specific context are addressed. This involves considering aspects such as ambient illumination, channel distance, symbol variability, and algorithm robustness against possible distortions or interferences.

### 2.1. Data Collection

**Figure 1:**
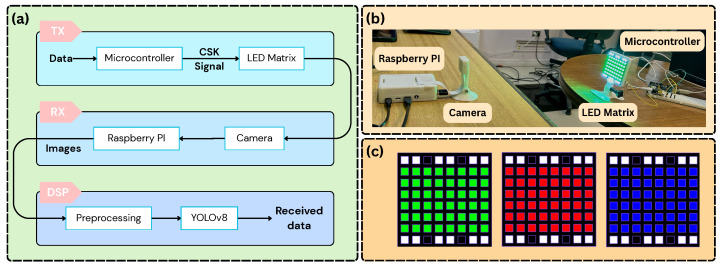
System architecture and experimental setup: (**a**) Corresponding block diagram illustrating the system components and data flow; (**b**) experimental setup used for data acquisition; (**c**) colors on the LED matrix.

The experimental setup used to generate the dataset is shown in Figure 1. It consists of a transmitter (LED matrix and microcontroller) and a receiver (camera and processing unit), as described below.

An LED matrix controlled using a microcontroller is used as a transmitter. As a modulation format, we used 8-Color Shift Keying (8-CSK) with colors: yellow, blue, white, cyan, magenta, orange, red, and green. This follows the IEEE 802.15.7-2011 standard [19], which sets design rules for the 8-CSK constellation to achieve reliable performance. In 8-CSK, every symbol carries 3 bits of information. All symbols used the same spatial arrangement in the LED matrix. To reduce the blooming effect, common in OCC, each symbol includes white LEDs at the top and bottom rows (see Figure 1c). In addition, the microcontroller applies gamma correction to adjust for the nonlinear behavior of the LEDs, using the following transformation on each RGB channel:

(1) $$C_{out}=255{\left(\frac{C_{in}}{255}\right)}^{\gamma },$$

where $C_{out}$ and $C_{in}$ represent the output and input intensity values of the red, green, and blue channels, respectively. These values are constrained to the range [0, 255]. In this work, the gamma correction factor is set to γ=3. Each transmission sequence consisted of consecutively displaying the eight color symbols on the LED matrix at a frequency of 10 Hz, with each symbol active for a fixed time duration. In our work, eight colors for CSK modulation were chosen to balance data rate and detection accuracy. While it is possible to further expand the color alphabet, this introduces inherent challenges, particularly in distinguishing between visually similar colors, as shown in Table 1.

The receiver was a V2 camera connected to a Raspberry Pi 4. This camera integrates a Sony IMX219 CMOS image sensor [20]. The camera was configured to record video at 30 frames per second (FPS), resulting in an oversampling factor of 1.5, considering a transmitter symbol rate of 10 Hz. The captured videos contain frames where each symbol is visible under different conditions.

Five-second videos of the symbol sequences were recorded under varied experimental conditions to introduce diversity in the dataset. The parameters adjusted in each experiment were:

- Channel distance: 50 cm, 100 cm, 150 cm, 300 cm.
- Camera exposure time: 500 μs, 1000 μs, 4000 μs, 6000 μs.
- Angle between TX and RX: aligned and unaligned matrix.
- Controlled light environment: dark room and illuminated room.

**Figure 2:**
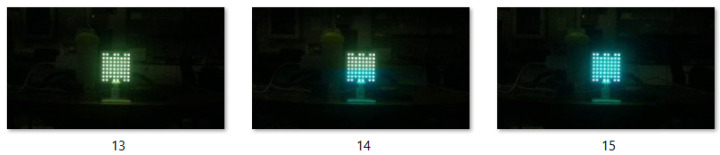
Frames captured from the cyan and magenta classes when oversampling.

Each video was processed frame by frame. Frames affected by symbol transitions or motion artifacts (such as frame 2 in Figure 2) were manually discarded, retaining only clean frames that represent each class [21]. Due to oversampling, an average of three usable images per class was extracted from each sequence. In total, 3,247 images were collected for training and validation. A separate test set was later acquired under the same parameter ranges, but at different random combinations of distance, angle, and exposure time within the laboratory. This ensured the test set included unseen samples that still followed the same distribution as the training data.

To provide a comprehensive overview of the experimental conditions, the key system parameters used for data collection are summarized in Table 2.

### 2.2. Data Preprocessing

Data pre-processing consists of four stages: data augmentation, dataset splitting, image resizing, and pixel value normalization. The data augmentation technique was applied directly to the raw data, i.e., to the data extracted from the captured videos, without having received any additional operation beyond the removal of non-useful frames. For this, the Albumentations library was used to first define the transformations and then apply them to all the collected images. In addition to these transformations, two additional components were incorporated: the addition of white Gaussian noise with different standard deviations (σ), and the superimposition of an office image on the images of the training, validation, and test sets.

**Figure 3:**
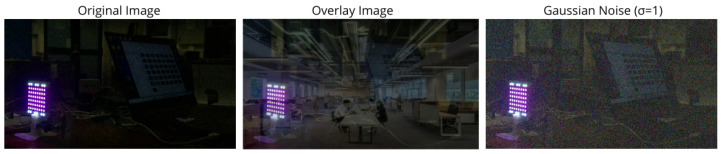
Transformations performed on the images in the test set.

Figure 3 illustrates the two transformations performed, which were applied randomly to the images. Thus, the training and validation samples were duplicated and, subsequently, one of three operations was applied: Albumentations transformations, noise addition (with mean 0 and a standard deviation randomly selected from the values (0.7, 1, 1.5, 2, 5), or office image overlay. All these operations have the same probability of being applied to images duplicated from the original data sets. The use of the overlay image was intended to increase the variability in the dataset, thereby enabling the model to learn to recognize the LED matrix in a wider variety of scenarios. This additional variability was necessary due to the exposure times used.

The samples intended for model evaluation were also duplicated; however, only the noise addition and image overlay operations were applied to them, excluding the Albumentations transformations, since these were used exclusively to improve model learning during training. The data splitting consisted of allocating 80% of the data for training and the remaining 20% for validation, from the 6494 samples obtained after data augmentation.

In the data resizing stage, a hyperparameter, known as *imgsz*, is set with the help of the YOLOv8 network, which is responsible for resizing the images to a predefined size. During the training of the deep learning model, YOLOv8 allows flexibility in the input sizes by automatically performing the resizing [22]. Normalization is an essential preprocessing technique that adjusts the pixel values of the images to a standard range, thus facilitating faster convergence during training and improving model performance. Normalization is automatically and seamlessly integrated as part of the preprocessing stage in YOLOv8 during model training. This automated preprocessing ensures that the input images are prepared consistently and properly before being processed by the CNN. To evaluate the balance of the generated and split dataset, the number of samples per class in each set is obtained, as shown in Table 3.

### 2.3. Model Selection and Training

To effectively address the challenge of classifying CSK symbols in real-world Optical Camera Communication (OCC) environments, this work employs a robust deep learning approach based on the YOLOv8 family of convolutional neural networks (CNNs), renowned for its exceptional balance between speed and accuracy. A central component of our methodology is the use of transfer learning: instead of training a model from scratch, which would require a vast amount of labeled data, we leverage a YOLOv8 model pre-trained on the large-scale ImageNet dataset. This enables the model to retain its powerful, generalized feature extraction capabilities while being fine-tuned for the specific task of CSK symbol classification. For our task, we adapted its main backbone, based on CSPDarknet, as a feature extractor and complemented it with a classification head capable of recognizing the eight color symbol classes in our dataset. This backbone integrates optimized convolutional modules, Cross Stage Partial layers for improved efficiency, and Darknet Bottleneck residual connections. Finally, the Spatial Pyramid Pooling Fast layer was replaced with a dedicated classification layer. This final layer transforms the extracted features into output predictions. Although originally designed to support up to 1000 output classes, the model automatically adjusts its final linear layer to 8 during training, matching the number of CSK symbol classes present in the dataset.

### 2.4. Validation and Adjustment of Hyperparameters

**Figure 4:**
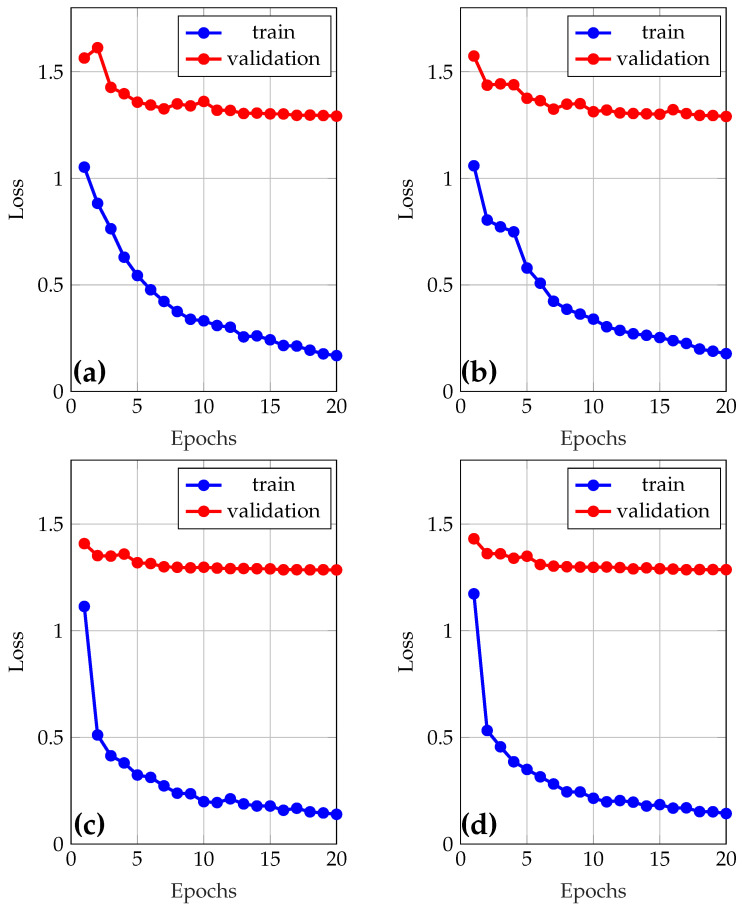
Learning and loss curves during model training. (**a**) Optimizer = AdamW, lr0 = 0.01, dropout = 0.0; (**b**) Optimizer = AdamW, lr0 = 0.01, dropout = 0.2; (**c**) Optimizer = AdamW, lr0 = 0.001, dropout = 0.0; (**d**) Optimizer = AdamW, lr0 = 0.001, dropout = 0.2.

A model was initially trained using the default hyperparameters of YOLOv8, with validation enabled. Subsequently, a manual tuning of the key hyperparameters such as the initial learning rate (lr0), optimizer choice (AdamW, SGD), and dropout rate was performed (see Table 4), seeking a balance between accuracy, stability, and generalizability. Two values per parameter were defined, and an exhaustive search was applied to train nine different combinations, including the model with default values. Comparing the results, it was observed that the model with adjusted hyperparameters does not represent an improvement compared with the default hyperparameters. Finally, the best model was retrained for 100 epochs to evaluate its performance robustly on the test set. Figure 4 shows the loss and the validation results during training of the models used to adjust the hyperparameters.

## 3. Results

This section details the performance of our YOLOv8-based classifier in the OCC system, beginning with a summary of the key parameters used to ensure reproducibility.

**Figure 5:**
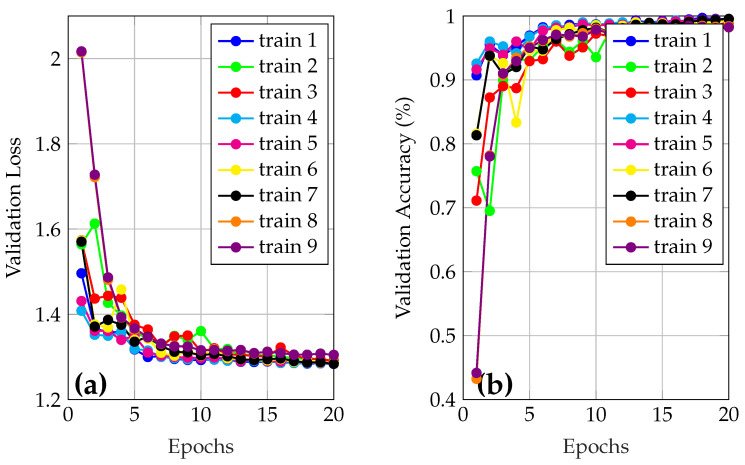
(**a**) Loss and (**b**) accuracy validation curves for trained models.

Table 5 presents the loss and accuracy results in the validation set for the various training runs performed during this process. This table facilitates the identification of the best hyperparameter settings for the classification task. In this table, the lowest loss value and the highest level of accuracy achieved during the epochs of the validation stage are presented. The validation curves (accuracy and loss vs epochs) for the reported data are shown in Figure 5.

In Figure 5a, the blue curve (train 1), corresponds to the best model and presents a low loss in most epochs, starting at 1.4961 and reaching a minimum of 1.2837. The curve shows a decline until the sixth epoch, where the loss stabilizes around 1.3. On the other hand, in Figure 5b, it is observed that the accuracy starts 90.71%, and achieves 99.69% after 20 epochs. This indicates, at first glance, that the model has outstanding performance even with a reduced number of iterations.

**Figure 6:**
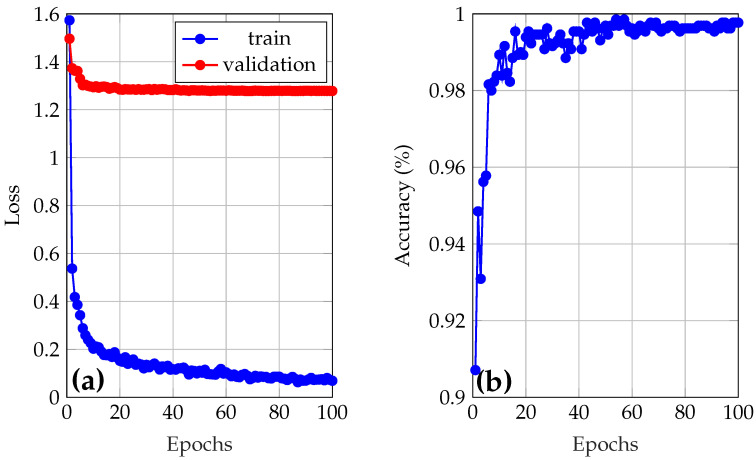
(**a**) Loss and (**b**) accuracy of the final model.

The best model from Figure 5 was selected (train 1), and was re-trained using the best hyperparameter configuration. This process generated the training and validation loss graphs, as well as the validation accuracy, which are shown in Figure 6a and Figure 6b, respectively. Training of the final model, minimum loss of 1.27 and maximum accuracy of 99.85% were achieved. This accuracy value will be used by YOLO as the criterion to select the best model, which will be used in the test set.

When analyzing the learning curves of the best model, trained for 100 epochs, in Figure 6a, a marked discrepancy between training loss and validation loss is observed. The training loss decreases rapidly, reaching very low values, while the validation loss remains significantly higher in comparison. However, it is seen that both losses could continue to decrease by further training the model using more epochs, suggesting that the model is not overfitting. Figure 6b shows a high validation accuracy throughout the epochs. The accuracy increases rapidly during the first epochs and stabilizes at values close to 98–99%. This high validation accuracy, combined with a high validation loss, could preliminarily indicate that the model makes correct predictions in most cases, but with low confidence in its classifications. That is, the model assigns lower probabilities to the correct classes, which is penalized by the loss function without affecting the overall accuracy. However, when analyzing the probabilities associated with each sample during validation, it was confirmed that this is not the case, as all samples were classified with reliabilities higher than 99%.

To evaluate the performance of the final model, a separate test set was used, different from the training and validation data. This set includes subsequently collected images covering various experimental conditions such as channel distances of 60 cm, 70 cm, 110 cm, and 250 cm, as well as different combinations of exposure time, environment variations, illumination levels, and transmitter positions. Additionally, these images were also processed to duplicate their quantity and. Noise and a different overlay image were added to the duplicated set.

**Figure 7:**
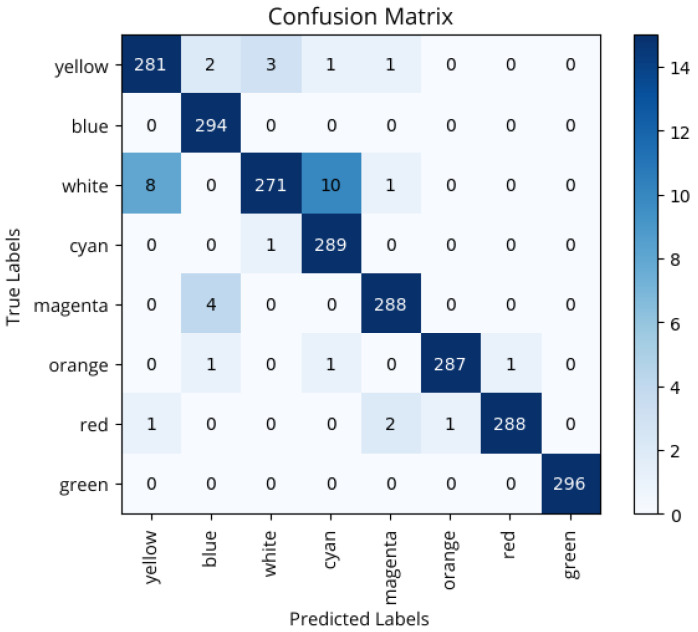
Confounding matrix before adding variability to the test set.

Predictions were made using the final model, with the results summarized in he confusion matrix shown in Figure 7. From the confusion matrix, it is observed that most predictions are concentrated on the main diagonal, indicating correct classification in the vast majority of cases. Classes such as green, blue, and cyan show near-perfect accuracy, with no errors or only one incorrect prediction. However, some specific confusions are observed, especially in the white class, which was misclassified as yellow in eight cases and as cyan in ten, suggesting some difficulty for the model in distinguishing between light spectrum colors or colors possibly influenced by similar lighting conditions. Minor errors are also recorded in other classes, such as yellow and magenta, although without significantly affecting the overall performance of the system.

It can be concluded that very good results are obtained, in general; however, some exceptions exist, especially among classes with visually similar samples, such as white and yellow, cyan and white, or blue and magenta. This is primarily due to the phenomenon of channel crosstalk, which causes interference between color channels as a result of spectral overlap in the LED array and the camera’s limitations to distinguish close tones. Additionally, factors like ambient lighting and noise in the capture system contribute to the camera registering color mixtures rather than pure tones, leading to confusion between these classes when interpreted by the YOLOv8 model.

The samples in each class were correctly classified with high accuracy and, overall, the model demonstrated a high generalization ability with an accuracy of 98.4% in the test set. The confusion matrix, therefore, not only confirms the strong overall performance of the model but also provides a nuanced understanding of its limitations. The strong concentration of correct predictions along the main diagonal, notably for classes like blue and green (with 294 and 296 correct predictions, respectively), demonstrates effective generalization under real testing conditions. However, the confusion between white, cyan, and yellow highlights the inherent physical constraints of the optical camera communication system, such as channel crosstalk and sensor saturation, when processing colors requiring high intensity across multiple RGB channels. Minor misclassifications, like magenta being confused with blue, further reinforce these.

The strong overall accuracy and robust performance of our model against varying distances and lighting conditions hold significant implications for its practical deployment. A symbol recognition accuracy of approximately 98.4% translates directly into an extremely low data transmission error rate, which is a critical requirement for safety-sensitive applications where reliability is paramount. In vehicle-to-vehicle (V2V) communication, for example, our system could be used to reliably classify emergency signals, such as the flashing lights of an ambulance or police car, or the red light of a traffic signal, providing a robust communication channel in complex and dynamic environments. Similarly, in industrial IoT scenarios, this high reliability is fundamental for the correct execution of machine-to-machine commands, preventing costly operational errors. Therefore, our results not only confirm the model’s technical efficacy but also validate its potential as a low-cost, high-reliability solution for real-world applications where low-data-rate, high-precision signaling is required.

In addition, system robustness in real-world applications could be further improved through specific design improvements. For instance, the use of light diffusers in vehicular and other outdoor scenarios would mitigate channel crosstalk, allowing color separation under conditions such as direct sunlight or car headlight interference. Similarly, adaptive gain control mechanisms in the camera receiver would allow automatic adjustment to varying illumination, thereby ensuring stable detection in dynamic outdoor environments. At the communication layer, advanced error correction coding could compensate for residual symbol misclassifications, while temporal and spatial filtering would stabilize detection in settings with strong motion or background variability. These enhancements, although not implemented in this work, are challenges observed in V2V and industrial IoT scenarios.

## 4. Conclusions

This paper evaluated the performance of the YOLOv8 model in symbol classification within optical camera communication environments. The results consistently demonstrated strong performance in all scenarios analyzed, achieving an accuracy of 98.4% on the test set, indicating the effective generalizability of the model. Incorporating noise transformations and varied data augmentation techniques applied to training and validation images was essential for developing a robust and resilient model. These strategies substantially improved generalizability, allowing the model to reach values above 93% across all evaluation metrics for each symbol class, even when the test set included noise and previously unseen samples.

From the outset, the model was designed and trained to handle diverse and complex conditions by simulating realistic environments during the training phase, which is essential for its successful deployment and practical application. Misclassified samples were primarily caused by channel crosstalk; specifically, the RGB color information captured by the image sensor is affected by overlapping signals from the red, green, and blue channels. This interference causes certain colors in the LED matrix, which should ideally appear as distinct hues (e.g., white), to be perceived as different colors, such as yellow or cyan. Such distortions highlight the inherent challenges of color-based modulation schemes in OCC systems.

For future research, two main directions are recommended. First, explore the implementation of the YOLOv8 model for real-time detection and sensing in OCC applications, which could offer valuable tools for automated monitoring and the management of low-latency optical communication systems. Second, developing a more diverse and comprehensive dataset is essential. This dataset should encompass variations such as different background textures, a wider range of channel distances, diverse ambient lighting conditions, and various indoor/outdoor scenarios, which will allow the model to generalize across a broader spectrum of real-world environments.

## Figures and Tables

**Table 1:** RGB values of the different classes.

Class	R	G	B
Yellow	255	255	0
Blue	0	0	255
White	255	255	255
Cyan	0	255	255
Mangenta	255	0	255
Orange	255	165	0
Red	255	0	0
Green	0	255	0

**Table 2:** Key System Parameters for Experimental Setup.

Parameter Category	Specifics
**Transmitter (TX)**	
LED Matrix	8×8 LED array
Modulation Scheme	Color-Shift Keying (CSK) with 8 levels
Transmission Frequency	10 Hz
**Receiver (RX)**	
Camera Type	CMOS V2 with Sony IMX219 sensor
Frame Rate	30 fps
**Channel & Environment**	
Communication Distance	30 cm to 3 m
Lighting Conditions	Dark room, illuminated room
TX-RX Alignment	Aligned and unaligned

**Table 3:** Number of samples per class in the datasets.

Class	Training	Validation	Test
0: Yellow	646	162	288
1: Blue	630	158	294
2: White	670	168	290
3: Cyan	657	165	290
4: Mangenta	646	162	292
5: Orange	664	166	290
6: Red	649	163	292
7: Green	630	158	296
Total	5192	1302	2332

**Table 4:** Hyperparameters to be adjusted in YOLOv8.

Hyperparameter	Default	Values	Definition
lr0	0.01	0.01, 0.001	Initial learning rate. Adjusting this value is crucial to the optimization process, influencing how quickly the model weights are updated.
optimizer	auto	AdamW, SGD	Choice of optimizer for training. Options include SGD, AdamW, etc. Affects convergence speed and stability.
dropout	0.0	0.0, 0.2	Dropout rate for regularization in classification tasks, preventing overfitting by randomly omitting units during training.

**Table 5:** Validation loss and accuracy results when training with different hyperparameter configurations.

Train	Optimizer	lr0	Dropout	Loss	Accuracy (%)
1	AdamW	0.000714	0.0	1.2837	99.69
2	AdamW	0.01	0.0	1.2918	99.69
3	AdamW	0.01	0.2	1.2902	99.00
4	AdamW	0.001	0.0	1.2850	99.39
5	AdamW	0.001	0.2	1.2862	99.46
6	SGD	0.01	0.0	1.2852	99.23
7	SGD	0.01	0.2	1.2838	99.54
8	SGD	0.001	0.0	1.3046	98.46
9	SGD	0.001	0.2	1.3049	98.46

## Data Availability

The original contributions presented in this study are included in the article. Further inquiries can be directed to the authors.
